# Ultrasound analysis of hemidiaphragm function in case of pleural effusion

**DOI:** 10.3389/fmed.2024.1532214

**Published:** 2025-01-17

**Authors:** Martin Boussuges, Fabienne Bregeon, Xavier Benoit D’Journo, Alain Boussuges

**Affiliations:** ^1^Service de Pneumologie, Centre Hospitalier Universitaire (CHU) de la Reunion Sud, Saint Pierre, France; ^2^Service d’Explorations Fonctionnelles Respiratoires, CHU Nord, Assistance Publique des Hôpitaux de Marseille, Marseille, France; ^3^Unité d’Appui à la recherche (HIPE), Aix-Marseille Université, CNRS, Université de Toulon, Institut Paoli-Calmettes, Marseille, France; ^4^Service de Chirurgie thoracique et des maladies de l’œsophage, CHU Nord, Assistance Publique des Hôpitaux de Marseille et Aix Marseille Université, Marseille, France; ^5^Centre de Recherche en Cardio-Vasculaire et Nutrition (C2VN), Aix Marseille Université, Marseille, France

**Keywords:** diaphragm, chest ultrasonography, dysfunction, normal value, anatomical M-mode

## Abstract

**Background:**

Diaphragm dysfunction is frequently observed in patients with pleural effusion. The aim of the study was to determine the criteria for estimating the impact of pleural fluid on diaphragm function and detecting impairment of diaphragmatic muscle.

**Methods:**

This was a retrospective observational study carried out in a university hospital. Cases of free pleural effusion were recruited from the ultrasound consultation of the lung function test laboratory. The quantification of pleural effusion and analysis of diaphragmatic function were performed using chest ultrasound performed while sitting. In case of abnormal diaphragmatic motion, the examination was repeated in supine position.

**Results:**

109 pleural effusions (57 left, 52 right) were included in the analysis. Pleural effusions were detected after thoracic surgery in 89% of cases and in the context of medical disease in other cases. Excursion during deep inspiration was reduced by the amount of fluid (4.3 ± 2.1 cm for small effusions, 3.2 ± 1.7 cm for moderate effusions and 1.1 ± 1.8 cm for large effusions). In 23 cases of large pleural effusion, the excursions during deep inspiration were always below the lower limit of normal. In some cases, a paradoxical motion suggesting hemidiaphragm paralysis was observed. When the inspiratory thickening was normal, the paradoxical excursions disappeared in supine position. In moderate pleural effusion (53 cases), hemidiaphragm excursion was above lower limit of normal in 68% of cases. In cases of paradoxical motions, repeated ultrasound examinations were in favor of hemidiaphragm paralysis. In small pleural effusion (32 cases) the excursion was most often normal.

**Conclusion:**

The ultrasound analysis of diaphragm excursion and thickening in sitting and supine positions is useful to assess the impact of pleural effusion and detect impairment in diaphragm muscle function.

## Introduction

Pleural effusion is characterized by an abnormal accumulation of fluid between the pleura layers. It can be observed after surgery or trauma and in various medical diseases (1). Most of the various circumstances and diseases that may be responsible for pleural effusion are also risk factors for phrenic nerve conduction impairment, leading to diagnostic difficulties.

Pleural effusion is frequently observed after thoracic surgery. In cardiac surgery patients, an effusion was reported in 37% of cases after coronary artery bypass grafting and in 25% of cases after valve surgery (2). In a recent study, fluid drainage was required in 59 out of 409 patients (14%) after coronary artery bypass grafting (3). Excess fluid in the pleural cavity is recognized as a factor affecting diaphragmatic function (4). In some cases, the dysfunction may mimick hemidiaphragm paralysis (5, 6). In addition, in cardiac surgery patients (7), the conduction of the phrenic nerve may be impaired through thermal lesion secondary to ice-cold solution in the pericardium or mechanical lesion during mammary artery dissection.

In medical patients, pleural effusion may be secondary to heart, liver or kidney failure, pulmonary embolism, infectious diseases or malignancy (8–13). In patients with heart failure, ablation of an atrial or ventricular arrhythmia may be necessary, exposing to phrenic nerve injury (14, 15). Infectious diseases such as COVID 19 can impair phrenic nerve conduction through inflammatory processes (16). In malignancy, diaphragm dysfunction may be secondary to direct compression of the phrenic nerve along its path (17).

In intensive care unit (ICU) patients, several factors may contribute to the high prevalence of pleural effusion, including the disease leading to admission to ICU and specific factors such as prolonged supine position, mechanical ventilation and decreased plasma oncotic pressure (18). Patients submitted to mechanical ventilation may develop intensive care unit-acquired diaphragm dysfunction (19, 20).

Therefore, many circumstances can combine pleural pleural effusion and true diaphragmatic dysfunction due to phrenic nerve damage or muscle weakness. Thoracic ultrasound is an accurate tool for diagnosing both pleural effusion and diaphragm dysfunction. The objective of this retrospective observational study was to determine predictive ultrasound criteria for the mechanism of diaphragm dysfunction, based on a study of a large sample of cases of pleural effusions.

## Methods

This retrospective observational study was carried out in a university hospital in Marseilles (France). This study fell within the French legal framework for non-interventional research as performed on medical data collected during standard clinical care. The study was reported to the health data access portal of the hospital institution and registered under reference HHPAF5/2024. The study was approved by the ethics committee of the French Society of Cardio-Vascular and Thoracic Surgery (decision: July 29th, 2024)^1^.

Cases of pleural effusion were recruited from the ultrasound consultation of the lung function test laboratory of the North Hospital. Patients were referred to the ultrasound consultation by medical doctors for various reasons such as abnormal chest X-Ray, or unexplained respiratory disorders. Medical charts were used to identify cases of pleural effusion detected by ultrasound. When paralysis or dysfunction of the hemidiaphragm was documented by ultrasound, additional information was sought, including fluid drainage, clinical status, and other methods of assessing diaphragm function such as repeated chest X-rays or phrenic nerve testing. In addition, as our team usually does, in case of abnormal diaphragmatic function, a follow-up visit including the same ultrasound examination procedure was scheduled. The results of such consultation were also sought in medical records. This retrospective study was an analysis of data that were recorded for reasons other than research and was classified as non-involving human subjects. In the medical consultation unit, all patients received a notice of information and non-objection according to French law. The study adhered to the ethical standards set out in the 2008 Helsinki declaration.

### Pleural effusion detection and quantification

During the ultrasound examination, pleural effusions were looked for on both sides in patients while sitting. To quantify pleural effusion, the pleural spaces were systematically screened by moving the ultrasound probe from the base to the apex of the thorax along the posterior axillary line. The quantification of pleural effusion was performed using the grading system proposed by Smargiassi et al. (21). This method was not validated for loculated pleural effusions, therefore free-flowing anechoic pleural effusions were retained, only. To quantify pleural effusion, the number of intercostal spaces through which it was possible to see the fluid, was counted. Pleural effusion quantity was classified as *small* (effusion seen in a single intercostal space), *moderate* (extension of pleural effusion to 2–3 intercostal spaces) and *large* (extension of pleural effusion to 4 or more intercostal spaces).

### Assessment of diaphragmatic function

The ultrasound examinations were performed by two experienced investigators (AB and MB). Diaphragmatic function was assessed using the recording of the excursion and the thickness of both hemidiaphragms. The ultrasound examinations were performed using a commercially available ultrasound machine (Vivid S60N, GE Medical System, Milwaukee, WI, USA) equipped with a cardiac probe (3Sc probe) for diaphragm excursion measurements and a linear vascular transducer (9 L probe) for diaphragm thickness measurements. The examinations were performed with patients in a sitting position.

#### Measurement of diaphragm excursions

**Figure 1:**
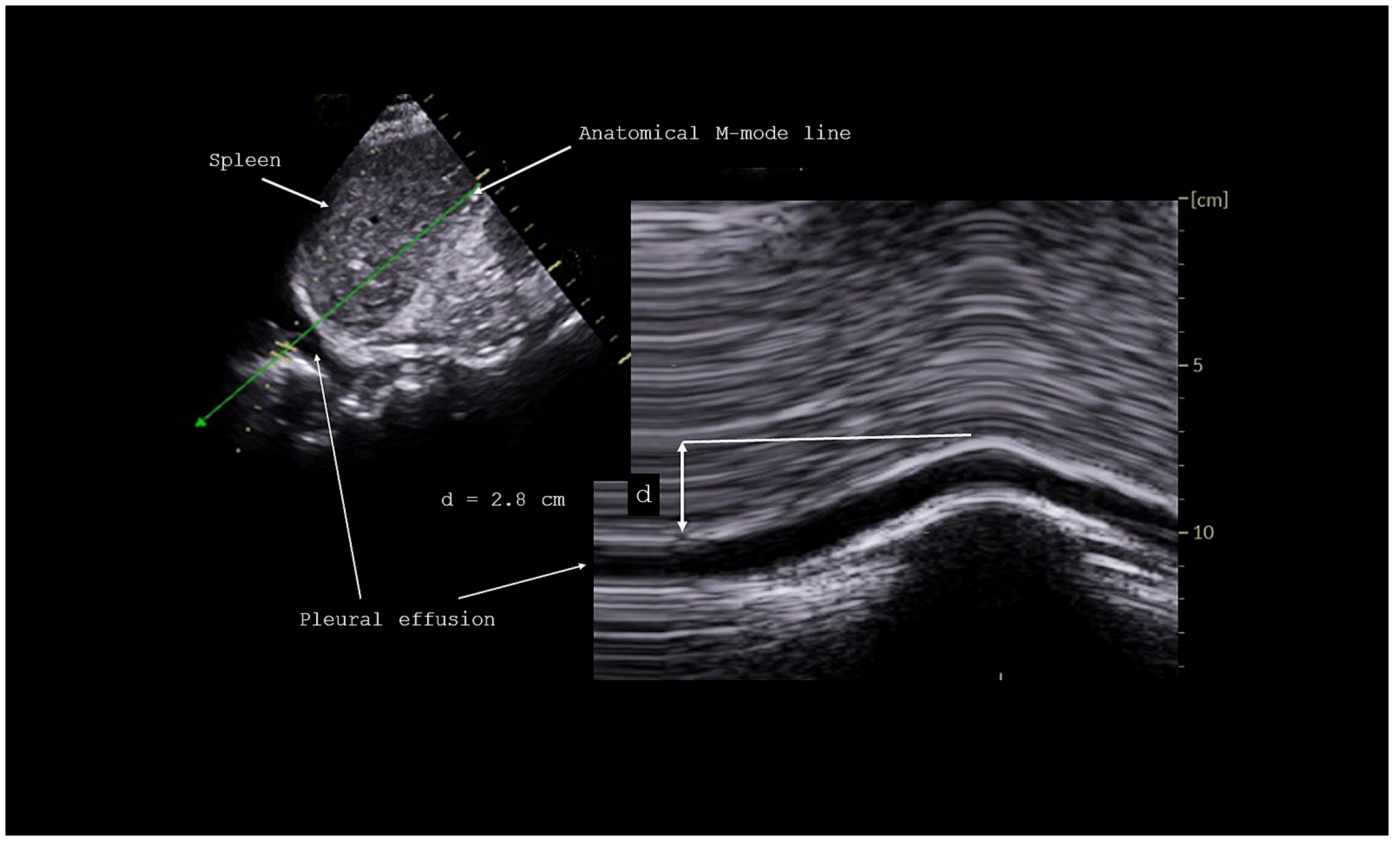
Measurement of diaphragmatic excursion during inspiration.

The excursions of both hemidiaphragms were measured using M-mode, as previously reported (22). Briefly, the probe was positioned on the subcostal or low intercostal area between the anterior and posterior axillary lines to visualize the right and left hemidiaphragms. The anatomical M-mode line was positioned to reach perpendicularly the posterior part of each hemi-diaphragm. Diaphragmatic motion was assessed under three conditions: during quiet breathing at tidal volume, during voluntary sniffing, and during deep inspiration at total lung capacity. After proper positioning of the calipers, the inspiratory diaphragm excursions were measured (Figure 1). Measurements were averaged from at least three different breathing cycles, except for deep inspiration, for which we selected the maximum excursion from several recorded maneuvers.

#### Measurement of diaphragm thickness

The right and left hemidiaphragms were visualized below the costophrenic recess near the anterior or the midaxillary line at the eighth or ninth intercostal space, where the diaphragm abuts the rib cage (apposition area) (23). The thicknesses of both hemidiaphragms were measured directly from the frozen B-mode images as the distance between the pleural membrane and peritoneal membrane, at the end of expiration and at the end of deep inspiration (24). The thickening fraction (TF) was calculated as the difference between the thickness at the end of deep inspiration and the thickness at the end of expiration, divided by the thickness at the end of expiration.

### Classification

#### Normal diaphragm function

When excursion and thickening measurements were above the lower limit of normal (LLN), based on recently published reference values (22, 25), hemidiaphragm function was classified as normal.

#### Abnormal diaphragm function

To detect abnormal diaphragm function, the excursions during deep inspiration had to be below the LLN, i.e., 3.3 and 4.1 cm on the right side and 3.2 and 4.2 cm on the left side, for women and men, respectively (22). In such patients, examination was repeated in supine position to assess whether the excursion increased above the LLN. The patient was therefore classified as normalized or not normalized in supine position. Paradoxical movements were sought during quiet breathing, voluntary sniffing and at the beginning of deep inspiration. If applicable, the impact of supine position on excursions was recorded. In addition, the thickening fraction was measured. Muscle impairment was diagnosed when TF was below 60% (25). Diaphragm dysfunction without complete paralysis was diagnosed when TF was between 20 and 60%. The detection of hemidiaphragm paralysis was based on recently published criteria (26). Briefly, a paradoxical movement of the hemidiaphragm was recorded during voluntary sniffing and at the beginning of deep inspiration. In addition, a non-significant thickening (less than 20%) or thinning should be observed to confirm paralysis.

### Statistical analysis

Results of the measurements were reported as Mean ± Standard Deviation. Statistical tests were run on Sigma Stat software (SPSS Inc., Chicago, USA).

Comparison of the ultrasound measurements according to pleural effusion quantity (small, moderate, large) was performed using one way ANOVA analysis. When the mean values between treatment groups reported a statistically significant difference, the multiple comparison procedure used a Tukey test. In cases of non-normal distribution, ANOVA on ranks was used. When treatment showed a significant difference, to isolate the group or groups that differ from others, the multiple comparison procedure used the Dunn method. The significance level was *p* < 0.05.

## Results

### Pleural effusions

126 cases of pleural effusion investigated by ultrasonography were initially screened, loculated pleural effusions were detected in 17 cases and were excluded. Finally, 109 pleural effusions (57 left, 52 right) were included in the analysis. Pleural effusions were detected by ultrasound 23 ± 12 days after thoracic surgery in 97 cases. Other medical conditions were heart failure, malignancy and infectious or inflammatory diseases in 12 cases.

### Diaphragmatic function

Table 1 reports the results of the ultrasound examination.

**Table 1:** Results of ultrasound examination.

	Pleural effusion		
	Small	Moderate	Large
Mesurement of excursion			
Normal excursion in sitting position	24	20	0
Normalization in supine position	1	16	7
When excursion abnormal in supine position			
Recording of thickening fraction			
Normal (>60%)	0	7	7
Decreased (>20, <60%)	5	4	8
Absent (<20%)	2	6	2
Total	32	53	24

In 23 cases of large pleural effusions, hemidiaphragm excursions during deep inspiration were always below LLN. After lying supine, the excursion reached LLN in 7 cases. Paradoxical movement was observed in sitting position in 10 cases. In 6 of these cases, TF was low. Paradoxical excursions disappeared in supine position in 7 cases and after pleural drainage in 1 case. In 2 cases, the paradoxical motion associated with a low TF remained unchanged during follow up in favor of hemidiaphragm paralysis.

In moderate pleural effusions (53 cases), hemidiaphragm excursion was normal when sitting in 20 cases (38%). In 16 cases (30%), it was decreased when sitting but increased above LLN when supine. In 11 cases, the excursion remained below LLN when supine with a decreased TF in 4 cases. In 6 cases, hemidiaphragm paralysis was detected.

In small pleural effusions (32 cases), hemidiaphragm excursions, studied while sitting or supine, were most often normal (25 cases). In cases of abnormal motion, ultrasound was in favor of diaphragm dysfunction in 5 cases and hemidiaphragm paralysis in 2 cases.

Table 2 shows the impact of pleural effusion on diaphragmatic motion as a function of its quantity.

**Table 2:** Diaphragmatic motion and amount of pleural effusion.

	Small	Moderate	Large	*p*-value
Inspiratory excursion (QB) (cm)	1.9 ± 0.8^*, §^	1.4 ± 0.7^£^	0.5 ± 0.8	<0.001*
Voluntary sniffing (cm)	2.2 ± 1.3^§^	1.7 ± 1.4^£^	0.4 ± 1.7	<0.001
Inspiratory excursion (DI) (cm)	4.3 ± 2.1^*, §^	3.2 ± 1.7^£^	1.1 ± 1.8	<0.001
Thickening fraction (DI) %	102 ± 77^§^	66 ± 50	46 ± 34	0.02

QB, quiet breathing; DI, deep inspiration.

Significant difference.

*small vs. moderate, ^§^small vs. large, ^£^moderate vs. large.

### Clinical information

Chest X-ray found a raised hemidiaphragm in 10 cases of paralysis detected by ultrasound. In these patients, the diagnosis of paralysis was supported by electromyography with no response to phrenic stimulation.

#### Diaphragmatic function after pleural effusion drainage

In 5 cases of large pleural effusion, breathlessness caused pleural drainage (3 on the left side, 2 on the right side). Excursion during deep inspiration was below LLN in all cases. In 3 cases, a paradoxical motion was recorded while sitting with a decreased thickening fraction in one case. In 2 cases, paradoxical excursions disappeared in supine position. In 1 case the hemidiaphragm paralysis profile (paradoxical motion and low inspiratory thickening) remained unchanged in supine position. After drainage, the paradoxical motion disappeared, and the thickening fraction was normalized in all cases.

### Follow up

When the first ultrasound showed a decreased excursion in seated position and no normalization in supine position (41 cases), a follow up visit could be performed in 30 cases, 17 ± 7 days after the first examination. There was a decrease in the amount of pleural effusion in 19 cases.

- Among the 10 cases of hemidiaphragm paralysis, follow-up was possible in 8 cases and persistent paralysis was observed in all cases.
- In 4 cases with signs of diaphragm dysfunction without complete paralysis, the ultrasound examination remained unchanged.
- In 11 cases with a decreased motion and a normal TF at the first ultrasound, the excursions increased during the control visit.
- In 7 cases with a large pleural effusion and a paradoxical motion in sitting position recorded by the first examination and a normal motion in supine position, the decrease in the amount of pleural effusion led to normalization of excursions regardless of position.

Table 3 presents the results of the ultrasound examination according to the medical setting.

**Table 3:** Ultrasound examination and medical setting.

Medical setting	Number of cases	Hemidiaphragm paralysis	Recent mechanical ventilation	Pleural effusion			Hemidiaphragm paralysis
		Diagnosed before US		Large	Moderate	Small	Diagnosis after US
Cardiac surgery CABG, valve surgery	71	1	8	17	37	17	5
Other thoracic surgery	26	1	9	4	11	11	5
Heart failure	7	0	0	3	4	0	0
Infectious disease	3	0	0	0	0	3	0
Inflammatory disease, malignancy	2	0	0	0	1	1	0

CABG, coronary artery bypass grafting; US, ultrasound.

Recent mechanical ventilation: more than 2 days within 1 month before ultrasound examination.

## Discussion

Improvements in the analysis of diaphragmatic function in patients with pleural effusion can be achieved through the results of this study.

In cases of significant pleural effusion, the excursion is usually limited while sitting. In our study, no excursion reached the lower limit of normal during deep inspiration. In addition, in 10 cases, paradoxical motions were recorded during the voluntary sniffing and at the beginning of deep inspiration, suggesting an hemidiaphragm paralysis (27). In some cases, this ultrasound profile disappeared in supine position.

Muscle length is a primary determinant of the pressure-generating capacity of the diaphragm. De Troyer et al. showed that the adverse effect of pleural effusion on the diaphragm was secondary to the reduction in muscle length (28). Due to the effects of gravity on abdominal contents and pleural fluid, for a given effusion, the diaphragm has a caudal displacement and is shorter in the head-up than in the supine posture, leading to further impairment of diaphragmatic function (29). This phenomenon probably explained the impairment of the hemidiaphragm motion in the head-up posture compared to the supine posture recorded by ultrasound in our study.

Recording the diaphragm excursion when changing from sitting to supine position should be considered as a simple and useful method for assessing pleural fluid impact and hemidiaphragm function quality. When pleural fluid is responsible for the hemidiaphragm paralysis profile, ultrasound examination in supine position may show an improvement in diaphragm motion such as the regression of paradoxical movements. In such cases, follow up in our study reported a recovery of normal diaphragm function after a decrease in fluid quantity.

As our case studies and statistical analysis show, pleural effusion also has an impact on inspiratory thickening. Decreased thickening fraction has already been reported in patients with pleural effusion compared to healthy controls (30). In case of suspicion of hemidiaphragm paralysis, in addition to the recording of the excursion in supine position, the analysis of the thickening fraction provided an additional argument on the quality of muscle function. In most cases, although paradoxical movements were recorded while sitting, it was possible to detect an inspiratory thickening in favor of the absence of complete paralysis.

Pleural drainage has been reported to reduce shortness of breath in patients with abnormal hemi-diaphragm movements (31). Diaphragmatic ultrasound might provide an additional argument for pleural drainage. In our population, the decision of drainage was made by clinicians who are responsible for patients and based on clinical condition. In 7 cases, large effusions with paradoxical movement while sitting were not evacuated because the respiratory status was not significantly impaired. The clinical history reported a decrease in fluid volume and recovery of normal diaphragmatic movement, except for two cases of severe hemidiaphragm dysfunction. Therefore, recording a paradoxical movement in a sitting position does not seem sufficient to decide on the drainage of a pleural effusion when the patient has no significant respiratory disorders.

In some cases, paradoxical movements in all positions and a decrease in inspiratory thickening may be observed. In such circumstances, it is impossible to determine whether the hemidiaphragm is paralyzed or simply impaired by the amount of fluid, and fluid drainage is the only way to assess the quality of hemidiaphragm muscle function. In the absence of respiratory disorders, repeated ultrasound examinations should be recommended to reassess the function of the hemidiaphragm after the decrease in fluid quantity.

In moderate pleural effusion, a large percentage of cases (38%) had normal hemidiaphragm motion. Decreased excursion during deep inspiration was observed in sitting position in 51% of the cases. The contribution of fluid in the pleural space to the decrease in diaphragmatic movement was supported by the measurement of excursion in the supine position with a normalization in 59% of them (16 out of 27). In some cases, the excursion increased but did not reach the lower limit of normal. In such cases, the recording of thickening fraction was informative. Indeed, patients with a normal thickening fraction showed normalization during follow-up. In contrast, the 6 cases of hemidiaphragm paralysis remained unchanged, suggesting that this profile could not be secondary to moderate pleural effusion.

In patients with a small amount of fluid in the pleural space, diaphragm motion was most often normal. Excursion at deep inspiration was normal in 24 of 32 cases and in 1 case the excursion reached LLN when the study was performed in supine position (in total 78%). If signs of diaphragm dysfunction such as diaphragm paralysis or limited excursion during deep inspiration are present, impaired muscle function should be suspected. To strengthen the diagnosis recording of the inspiratory thickening is useful. The absence of significant thickening or thinning is in favor of impaired diaphragm function.

The impact of pleural effusion on clinical condition and diaphragmatic function is not only secondary to the amount of fluid. The change in chest wall mechanics seems to play a role (28, 32). Various factors such as thoracic volume, rib cage compliance, pulmonary status and diaphragmatic muscle quality could explain the high variability in the impact of pleural effusion on diaphragmatic motion observed in our study, particularly when the amount of fluid was large or moderate. Therefore, the search for signs of poor respiratory tolerance such as polypnea, hyperactivity of accessory inspiratory muscles and low transcutaneous oxygen saturation remains important to decide on the interest of evacuating pleural fluid.

**Figure 2:**
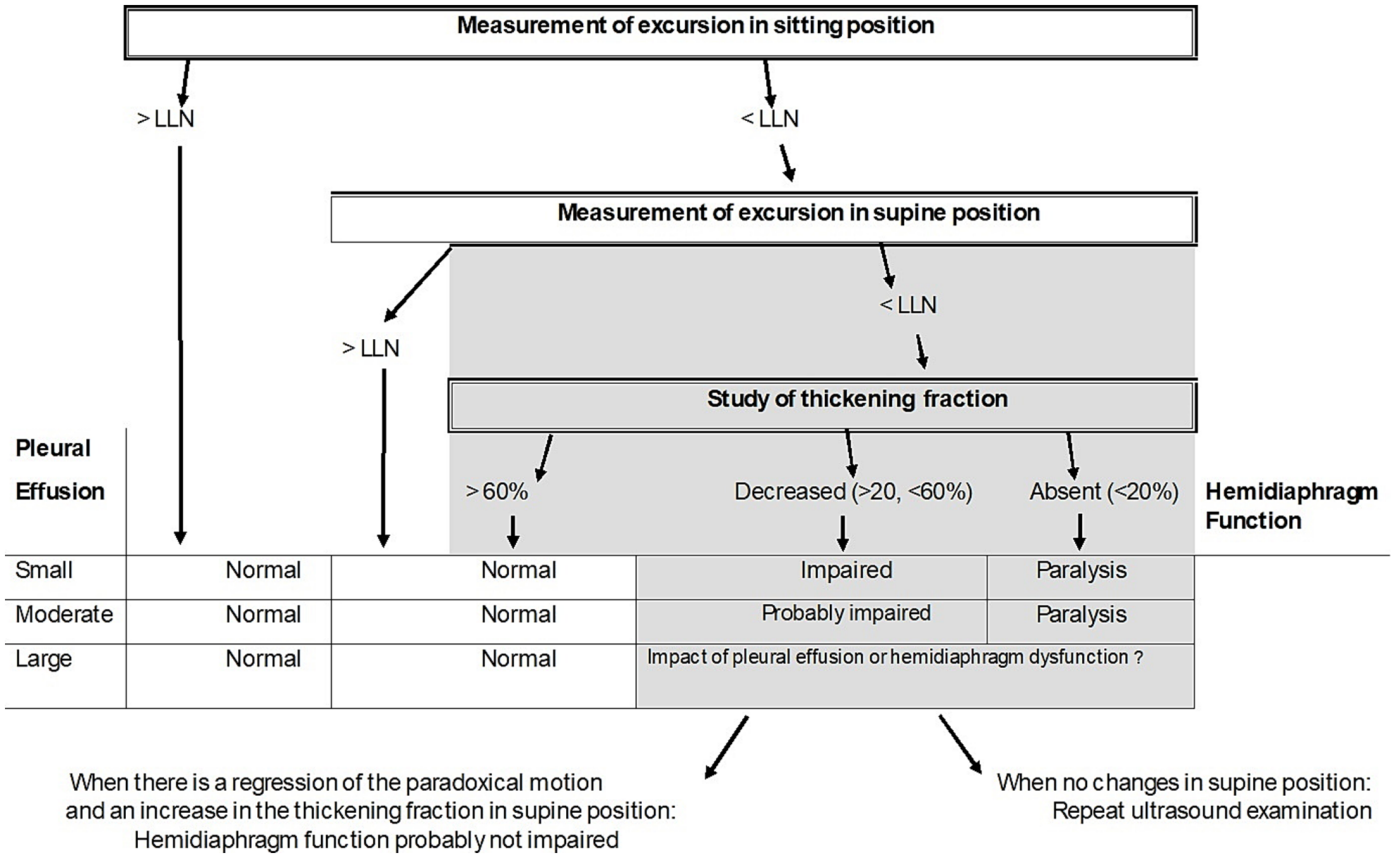
Proposed algorithm to estimate the quality of diaphragm muscle function in case of pleural effusion. LLN = Lower limit of normal.

According to our results, an algorithm based on ultrasound recording of the excursion and thickening of the diaphragm may be proposed to study hemidiaphragmatic function in patients with pleural effusion (Figure 2).

### Study limits

The grade used to estimate fluid in pleural effusion was designed for free-fluid effusions, therefore loculated effusions were not included in our study. The impact of changes in body position in loculated effusion is uncertain. Therefore, further studies on this topic would be useful. In our retrospective study, the detection of pleural effusion was most often performed after surgery. As our study was designed to analyze hemidiaphragm function, this was useful because the risk of diaphragmatic dysfunction was high in these patients (33). On the other hand, pleural effusion was rarely found in the context of medical illness (12 cases out of 109). To support our results and assess the accuracy of the algorithm proposed, it would be interesting to carry out a prospective study and explore more pleural effusions induced by medical diseases. It is recognized that diaphragm dysfunction is most often reversible in the weeks or months following phrenic nerve injury (34, 35). In our study, while the amount of pleural fluid decreased, an improvement in diaphragm muscle function between the first ultrasound and follow-up could not be excluded.

## Conclusion

Combining the measurement of excursion and thickening of the diaphragm in sitting and supine position allows to assess the impact of pleural fluid on diaphragmatic motion and detect impaired diaphragm muscle function. When the amount of pleural fluid is large, a hemidiaphragm paralysis profile can be observed. The regression of paradoxical movement in supine position, associated with the detection of significant inspiratory thickening is in favor of unimpaired diaphragm muscle function. When pleural drainage is needed, ultrasound monitoring is useful. In moderate pleural effusion, a decrease in pleural fluid-induced excursion is frequently observed. In contrast, when a hemidiaphragm paralysis profile is found, an impairment in diaphragmatic function may be expected. In small pleural effusion, when the muscle function of the hemidiaphragm is preserved, the excursion measured during deep breathing should reach the lower limit of normal.

## Data Availability

The datasets generated for this study are available on request from the corresponding author.

## Footnotes

^1^ https://www.sfctcv.org
